# Selection of Atoxigenic *Aspergillus flavus* for Potential Use in Aflatoxin Prevention in Shandong Province, China

**DOI:** 10.3390/jof7090773

**Published:** 2021-09-18

**Authors:** Jia Xu, Peng Wang, Zehua Zhou, Peter John Cotty, Qing Kong

**Affiliations:** 1 School of Food Science and Engineering, Ocean University of China, Qingdao 266003, China; Xuj970719@163.com (J.X.); wangp960517@163.com (P.W.); cottypj@gmail.com (P.J.C.); 2 Food Technology Department, Wageningen University & Research, 6700 AK Wageningen, The Netherlands; zehua.zhou@wur.nl

**Keywords:** atoxigenic strains, *Aspergillus flavus*, aflatoxin contamination, competitive inhibition

## Abstract

*Aspergillus flavus* is a common filamentous fungus widely present in the soil, air, and in crops. This facultative pathogen of both animals and plants produces aflatoxins, a group of mycotoxins with strong teratogenic and carcinogenic properties. Peanuts are highly susceptible to aflatoxin contamination and consumption of contaminated peanuts poses serious threats to the health of humans and domestic animals. Currently, the competitive displacement of aflatoxin-producers from agricultural environments by atoxigenic *A. flavus* is the most effective method of preventing crop aflatoxin contamination. In the current study, 47 isolates of *A. flavus* collected from peanut samples originating in Shandong Province were characterized with molecular methods and for aflatoxin-producing ability in laboratory studies. Isolates PA04 and PA10 were found to be atoxigenic members of the L strains morphotype. When co-inoculated with *A. flavus* NRRL3357 at ratios of 1:10, 1:1, and 10:1 (PA04/PA10: NRRL3357), both atoxigenic strains were able to reduce aflatoxin B_1_ (AFB_1_) levels, on both culture media and peanut kernels, by up to 90%. The extent to which atoxigenic strains reduced contamination was correlated with the inoculation ratio. Abilities to compete of PA04 and PA10 were also independently verified against local aflatoxin-producer PA37. The results suggest that the two identified atoxigenic strains are good candidates for active ingredients of biocontrol products for the prevention of aflatoxin contamination of peanuts in Shandong Province.

## 1. Introduction

Aflatoxins are a group of toxic, carcinogenic, secondary metabolites produced primarily by members of the *Aspergillus* section Flavi [1]. Exposure to even small concentrations of aflatoxins may lead to immunosuppression, cancer, and stunted growth, while ingestion of high concentrations may result in acute symptoms including hepatitis, liver necrosis, and death [2]. From 2004 to 2006, a severe outbreak of acute aflatoxin poisoning occurred in Kenya, resulting in 317 cases with a 39% fatality rate [3]. The major classes of aflatoxins are aflatoxin B_1_ (AFB_1_), aflatoxin B_2_ (AFB_2_), aflatoxin G_1_ (AFG_1_), and aflatoxin G_2_ (AFG_2_), of which AFB_1_ is the most common and toxic. Typically, most strains of *A. flavus* produce only B aflatoxins [4]. Contamination usually begins during crop development when the crop is associated with aflatoxin-producing fungi. Contamination may continue and become much more severe after the harvest if crop and storage conditions are not optimal. Aflatoxins may cause serious food and feed safety problems worldwide, resulting in serious economic losses [5]. As one of the host crops most susceptible to aflatoxin contamination, peanuts have been plagued by perennial contamination problems [6]. The ability of different strains of *A. flavus* to produce aflatoxin varies greatly, and in the natural environment, genotypes that produce aflatoxins are present at the same time as genotypes without the capacity to produce aflatoxins [7,8,9]. Generally, *A. flavus* populations are divided into two morphotypes based on sclerotia size: S strains (sclerotia diameter < 400 μm), L strains (sclerotia diameter > 400 μm) and physiology. The S and L strain morphotypes produce significant differences in the number of sclerotia and spores, and on average, S strain genotypes produce higher levels of aflatoxins [10,11,12,13]. In addition, genotypes also vary in ability to produce cyclopiazonic acid (CPA) a less toxic secondary metabolite [14]. Currently, researchers were divided on whether active ingredients capable of producing CPA should be considered for biocontrol products [15,16].

In addition to effects on the health of humans and domestic animals, aflatoxins are a detriment to agricultural economies. Aflatoxins in crops can lead to loss of markets and even crop destruction. Expenses for monitoring and decontamination can also be significant [17]. Therefore, it is desired that farmers effectively prevent the formation of aflatoxins in crops. The use of fungicides, timely irrigation, and alternate planting systems have shown limited effects in preventing aflatoxin contamination [18], and the safety and cost/benefit balance of these procedures are still unclear [18,19]. At present, the use of native atoxigenic strains endemic to target areas is the most effective tool for preventing contamination. Through these techniques, aflatoxin-producers are displaced by atoxigenic strains during competition throughout treated fields, resulting in lower frequencies of aflatoxin-producers and corresponding reductions of aflatoxins in crops [20]. The biological control products containing atoxigenic strain as an active ingredient can reduce the aflatoxin content of cash crops by 70–99% [21].

From Arizona cottonseed, AF36, the first and longest registered atoxigenic *A. flavus* (the active ingredient) is used on corn, cottonseed, almonds, figs, and pistachios [22]. AF36 is unable to produce the prerequisite substances necessary for aflatoxin formation due to a defect in the aflatoxin biosynthesis gene *pksA*, but it has an intact CPA biosynthesis gene cluster [23,24]. In Georgia peanuts, *A. flavus* NRRL21882, the active ingredient of the biocontrol agent Afla-Guard^®^, was isolated with deletion of the entire aflatoxin and CPA biosynthetic gene cluster. Afla-Guard^®^ is used to manage aflatoxin contamination in peanuts and corn [25,26]. Although, biocontrol strains have been increasingly adopted worldwide, work on biocontrol needs to be expanded to determine the most cost-effective manner to implement this technology to improve human well-being, the viability of agriculture, and environmental health. As more biocontrol programs are adopted globally to reduce aflatoxin contamination, strains used as biopesticides should be sourced from local fungal populations to reduce the risk of unpredictable ecological outcomes of non-native introduced organisms [27,28].

At present, China is the world’s largest peanut producer [29], and Shandong is one of the four major peanut-producing provinces in China. In Shandong, aflatoxin contamination is a central concern. However, biocontrol products with atoxigenic *A. flavus* active ingredients are not available in Shandong. The current study sought to identify atoxigenic *A. flavus* associated with peanut production in Shandong and to assess the potential of the fungi as active ingredients of biocontrol products directed at limiting aflatoxin contamination. During the process, two atoxigenic genotypes of *A. flavus* with the capacity to reduce aflatoxin contamination of peanut were cultured from Shandong peanuts. These two *A. flavus* genotypes have potential as active ingredients in biocontrol products directed at preventing aflatoxin contamination in the vital peanut production region of China’s North Central Plain.

## 2. Materials and Methods

### 2.1. Fungal Isolates and Culture Conditions

Two peanut samples from Shandong Province were crushed, suspended in sterile water (10 g in 90 mL), and subjected to dilution plate technique on CU medium as modified by Kachapulula et al. [2]. After incubation of plates at 30 °C for 3–5 d members of *Aspergillus* section *Flavi* were transferred to 5/2 medium (5% V8 vegetable juice, 2% agar, pH 5.2) and incubated for 5 days at 30 °C under dark conditions. Cultures were then stored as plugs of sporulating cultures in glass vials containing sterile distilled water.

Two aflatoxin-producers were used in competition experiments designed to identify *A. flavus* with potential value as biocontrol agents directed at preventing aflatoxin contamination. NRRL3357 was the standard aflatoxin producer. Isolate PA37 was selected as a local aflatoxin producer from Shandong peanuts to ensure competitiveness against local causal agents of contamination.

### 2.2. Characterization of Atoxigenic A. flavus

#### 2.2.1. Morphological Identification

Sporulating colonies were picked from 5/2 medium and were stored in brown strain preservation bottles containing 1/3 volume of sterile water at room temperature. Mature mycelia were selected and placed on slides, sections were made by adding 0.85% saline dropwise, and observed under a light microscope (10 × eyepiece, 40 × objective). Isolates were incubated on Wickerham medium as modified by Chang et al. [26] at 30 °C for 14 days after which conidia were washed from colonies with 95% ethanol, discarded, and formation of sclerotia on the agar surface was observed [30].

#### 2.2.2. Molecular Identification

Genomic DNA was extracted with omega DNA Fungal DNA Kit (D3390, Omega Bio-Tek, Georgia, USA) and molecular identification of the strains to be tested was performed using fungal universal primers (ITS1: TCCGTAGGTGAACCTGCGG, ITS4: TCCTCCGCTTATTGATATGC). The PCR products were sent to Qingdao Rui-Biotech Co., Ltd. (Qingdao, China) for sequencing, and the sequencing results were blasted in NCBI.

#### 2.2.3. Cluster Amplification Patterns

Cluster amplification pattern (CAP) is a rapid multiplex PCR method used to identify and monitor indels in the aflatoxin biosynthesis gene cluster and adjacent regions [31]. The CAP method was slightly modified from Callicott et al. [31]. Genomic *A. flavus* DNA was used as templates for PCR reactions using the primers and panel mixes from Callicott et al. [31]. DNA of *A. flavus* NRRL3357 served as the standard in all reactions. Reaction system: 0.8 μL Panel Mix, 3 μL DNA template, 10 μL 2× PCR Mix, sterile water to 20 μL total. Reaction conditions: pre-denaturation 94 °C, 1 min, then 30 cycles of denaturation 94 °C, 30 s, annealing 62 °C, 90 s, extension 72 °C, 90 s for 30 cycles, followed by a final extension of 72 °C for 10 min. PCR products were stored at 4 °C until results were visualized following 1% agarose electrophoresis.

#### 2.2.4. Aflatoxin Content in YES Media

Aflatoxin was extracted with methanol from seven-day cultures (30 °C stationary incubation) in YES media. The cultures containing methanol were shaken on an orbital shaker for 1 h at 200 rpm and 30 degrees. The methanol culture mixtures were placed in a centrifuge at 3500× *g* for 15 min. Supernatants were applied to aflatoxin purification columns and 50 μL of the effluent was subjected to high-performance liquid chromatography with a Hewlett-Packard Model 1100 pump (Palo Alto, CA, USA) connected to a Hewlett-Packard Model 1046A programmable fluorescence detector and a Hewlett-Packard Kayak XA (Hewlett-Packard Chemistry Station version 06.01) data module. Chromatographic separation was performed on a C18 reversed-phase column (150 mm × 4.6 mm ID, 5 μm particle size; Luna-Phenomenex, Torrance, CA, USA) connected to a pre-column safety guard (20 mm × 4.6 mm ID, 5 μm particle size, Phenomenex). The mobile phase was water: methanol: acetonitrile (4:1:1, *v*/*v*) with a flow rate of 1.5 mL/min and detection limits of 0.1 ng/g AFB_1_ and AFG_1_ and 0.03 ng/g AFB_2_ and AFG_2_. Comparisons were made to known aflatoxin standards (Sigma Aldrich, St. Louis, MO, USA).

#### 2.2.5. CPA Content Determination

Isolates PA04 and PA10 were incubated on Wickerham medium as modified by Chang et al. [26] for 7 days at 30 °C, and CPA was extracted with trichloromethane at 30 °C on an orbital shaker 200 rpm, for 30 min. The extract was passed through a Whatman 1 filter paper and concentrated by evaporation at room temperature. CPA concentrations were determined after thin-layer chromatography (TLC) on silica Gel G plates (105554) following the method of Chang et al. [26].

### 2.3. Competitive Analysis

Isolates PA04, PA10, PA37, and *A. flavus* NRRL3357 were incubated on YES medium at 30 °C for seven days, and the spores were washed off the plates with 0.01% Tween-80 to make a spore suspension. Suspensions were mixed with a vortex and spores were counted with a hemocytometer under a light microscope. Spore suspensions were diluted to 10^7^ spores/mL. The concentration of spore suspensions was diluted so that the final concentrations of PA04 and PA10 were 10^4^ spores/mL, 10^5^ spores/mL, and 10^6^ spores/mL. *A. flavus* NRRL3357 and PA37 spore suspension were diluted to a final concentration of 10^5^ spores/mL. Three mix ratios of 1:10, 1:1, and 10:1 (PA04:NRRL3357/PA37; PA10:NRRL3357/PA37) of *A. flavus* spore suspension were prepared.

#### 2.3.1. Competition of *A. flavus* on Peanuts

Peanut kernels were surface-sterilized by soaking in 75% ethanol for 3 min and rinsing with sterile water. 10 g of surface-sterilized peanuts per 250 mL conical vial were inoculated with 1 mL of each of the mixed conidial suspensions described above. Flasks were gently shaken to evenly coat the peanut kernels with a final water content of 90%. Peanuts inoculated with *A. flavus* NRRL3357/PA37 were the control treatments. Treatments were replicated three times. The conical vials were sealed with a breathable film to prevent contamination and incubated stationary at 30 °C and 85% humidity. AFB_1_ was quantified after 7 days.

#### 2.3.2. AFB_1_ Content of Inoculated Peanut

After seven days, *A. flavus* inoculated kernels were ground and one gram of ground peanut was added to 10 mL of methanol and shaken on an orbital shaker at 30 °C, 200 rpm, for 1 hour. Extracts were centrifuged at 3500× *g* for 15 min. The grinder was cleaned with ethanol to remove residues between samples. The supernatant was concentrated to 0.1 mL and subjected to a semi-quantitative aflatoxin analysis using TLC. TLC plates (Merck, Darmstadt, Germany) were developed in methanol: ethyl acetate: acetic acid (96:3:1) and air-dried. Aflatoxin was visible under 365 nm UV light. Extracts verified to have AFB_1_ by TLC were analyzed using an ELISA kit for aflatoxin B_1_ (Jiangsu Huisi Technology Co., Ltd., Jiangsu, China). The excitation and detection wavelengths were set at 360 nm and 450 nm, respectively. Inhibition of aflatoxin biosynthesis was expressed as an inhibition ratio (A) and was calculated using Equation (1).

A = [1 − C_2_/C_1_] × 100% (1)

where:

C_1_ is the concentration of aflatoxin B_1_ in the sample, and C_2_ is the concentration of aflatoxin B_1_ in control.

#### 2.3.3. Competition of *A. flavus* on Culture Media

The mixed conidial suspension (30 μL) spread evenly across PDA plates were compared with plates spread with NRRL3357 alone as a control. Each treatment was performed with three replicates, and cultures were grown in the dark at 30 °C for 10 days. Methanol was used to wash all cultures on the surface of the medium. The cultures containing methanol were shaken on an orbital shaker for 1 h at 200 rpm and 30 degrees. The methanol culture mixtures were placed in a centrifuge at 3500× *g* for 15 min after which AFB1 content was determined by TLC and ELISA kit.

#### 2.3.4. Statistical Analysis

The data were processed by SPSS 25 software. Treatments in all tests were replicated 3 times and means were subjected to Analysis of Variance and when significant differences were found, means were separated with Tukey’s HSD test.

## 3. Results

### 3.1. Characterization of A. flavus

**Figure 1:**
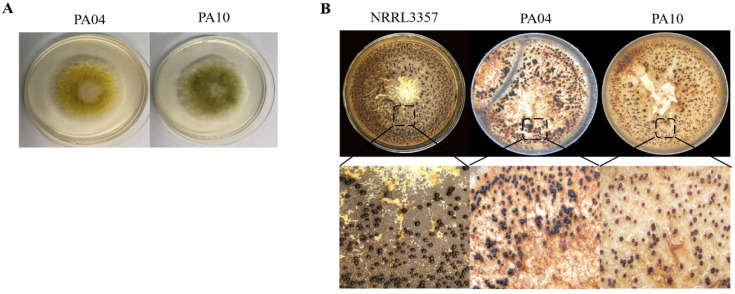
*Aspergillus flavus* PA04 and PA10 colonies incubated on Wickerham medium at 30 °C for 5 d (**A**) and sclerotia from cultures incubated at 30 °C for 14 d (**B**).

As shown in Figure 1, the colonies were initially flat, fluffy, and light yellow-green in color, and the center of the colonies was slightly elevated and slowly turned dark green in late maturity. Fungi with round or ellipsoidal vesicles with smooth spherical conidia were observed under 400×. Sequencing of PCR products obtained with the ITS universal fungal primers indicated that both PA04 and PA10 are more than 99% identical to *A. flavus*. The sclerotia of *A. flavus* PA04 and PA10 had average sclerotial diameters of >400 μm indicating both genotypes belong to the L strain morphotype.

CAP analysis of two strains of *A. flavus*, PA04, and PA10, revealed different numbers of bands missing in Panel2, with PA04 missing fragment regions of AC06 and IC02, and PA10 missing fragment regions of AC06, AC07, and IC02 (Figure S1). These band patterns indicate missing aflatoxin biosynthesis genes. In addition, both strains PA04 and PA10 had a large number of missing bands in the CAP assay Panel 3 and Panel 4. Panel 3 and Panel 4 amplify portions of the CPA biosynthetic gene cluster. The results of CAP analysis indicated that the two strains, PA04 and PA10, lack the genes necessary to produce aflatoxins.

**Figure 2:**
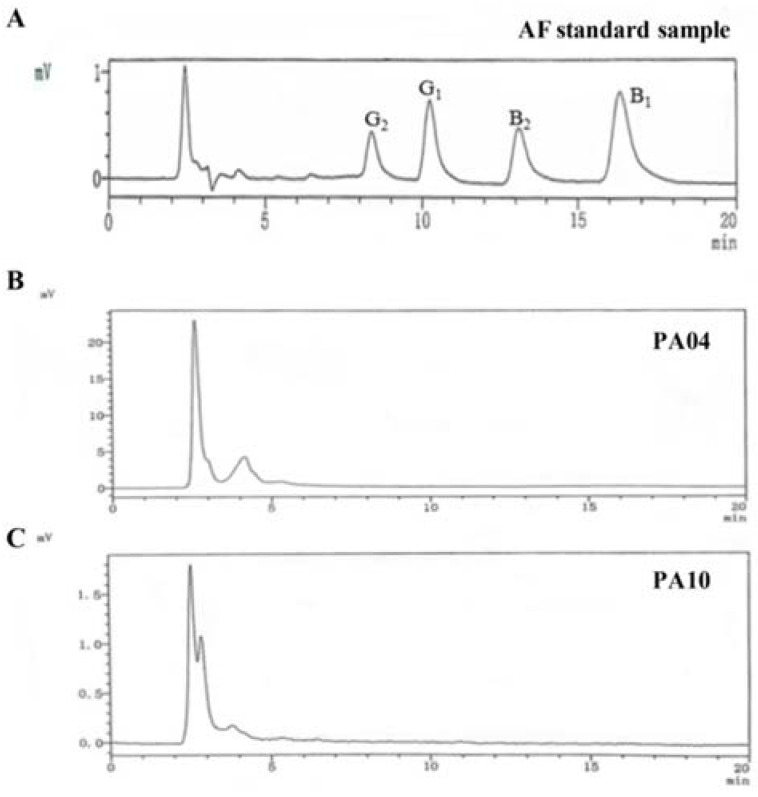
High performance liquid chromatography (HPLC) chromatogram indicating aflatoxin standard sample (**A**) and lack of aflatoxin production on YES medium (30 °C, 7 d) by both *A. flavus* PA04 (**B**) and PA10 (**C**).

Furthermore, PA04 and PA10 were found to not produce aflatoxins at the minimum detection limit (<0.1 ng/mL) for the HPLC protocol (Figure 2) confirming that the lack of detectable aflatoxin biosynthesis genes resulted in PA04 and PA10 lacking the ability to produce aflatoxins. The amount of CPA produced by *A. flavus* was determined by TLC and it was found that PA04 and PA10 produced small quantities of CPA compared to *A. flavus* NRRL3357(Figure S2).

### 3.2. Inhibition of Aflatoxin Biosynthesis by Atoxigenic Genotypes PA04 and PA10

**Figure 3:**
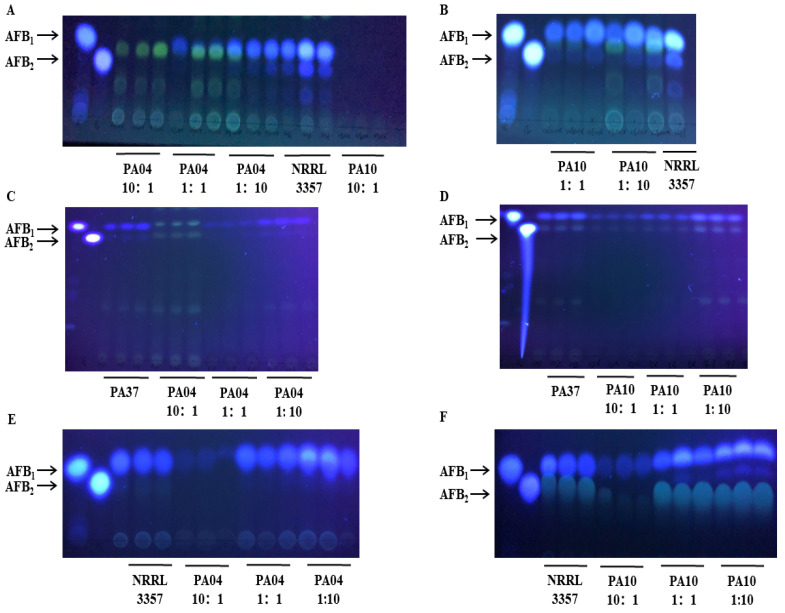
Thin layer chromatographs visualized under 365 λ UV light for aflatoxin extracts from co-inoculation experiments. Aflatoxin producer NRRL3357 alone and co-inoculated with three concentrations of either PA10 or PA04 and incubated at 30 °C for seven days on peanut (**A**,**B**) and 10 d on PDA medium (**E**,**F**). Aflatoxin producer PA37 alone and co-inoculated with three concentrations of either PA10 or PA04 and incubated at 30 °C for seven days on peanut (**C**,**D**).

**Figure 4:**
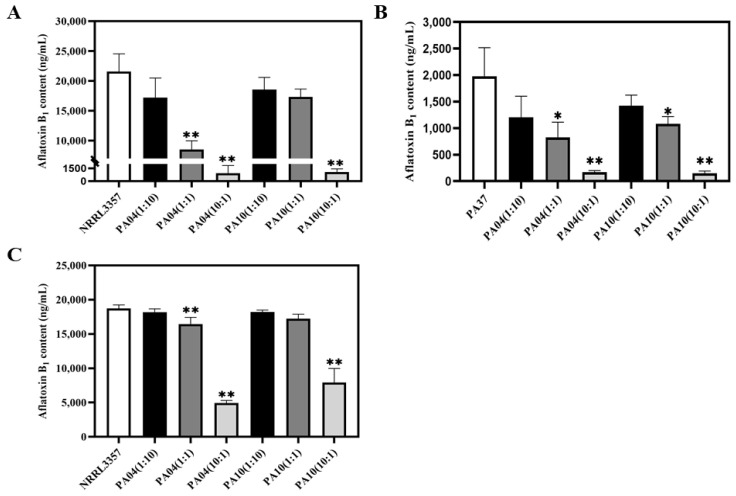
AFB_1_ concentrations as determined by ELISA resulting from co-culture of ei ther atoxigenic strains with NRRL3357/PA37. Peanut inoculated with different proportions of *A. flavus* strains and incubated at 30 °C for seven days (**A**,**B**). PDA media inoculated with different proportions of *A. flavus* strains and incubated at 30 °C for 10 days (**C**). (* *p* < 0.05, ** *p* < 0.001).

**Figure 5:**
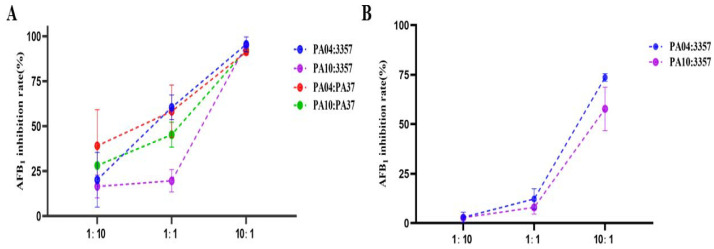
Inhibition of AFB_1_ biosynthesis by NRRL 3357 or PA37 with co-inoculation of different proportions of atoxigenic strains on peanuts at 30 °C for seven days (**A**). Inhibition of AFB_1_ production by NRRL 3357 with different inoculation ratios cultured on PDA at 30 °C, 10 d (**B**).

After seven days of incubation, peanuts inoculated with all three spore mixtures had significantly fewer aflatoxins than peanuts inoculated with *A. flavus* NRRL3357 alone. The lowest aflatoxin concentrations were in peanuts inoculated with PA04 and NRRL3357 at a 10 to 1 ratio (Figure 3). As the percent of atoxigenic strain in the inoculum increased, the aflatoxin content decreased (Figure 4). In addition, the AFB_1_ content of different inoculation ratios was measured using the Elisa method (Table S1). When the inoculation ratio was 10:1 (PA04:NRRL3357), the lowest AFB_1_ content was 923.1 ng/mL (Figure 4), and the AFB_1_ inhibition rate was 95.72% (Figure 5). When the inoculation ratio was 1:10 (PA04:NRRL3357), AFB_1_ inhibition was 20.18%, and its AFB_1_ content was 17,209.6 ng/mL. When co-inoculated with PA37, more obvious competitiveness was obtained. When the inoculation ratio was 10:1 (PA04:PA37), the lowest AFB_1_ content was 171.7 ng/mL (Figure 4), and the AFB_1_ inhibition rate was 91.31% (Figure 5). When the inoculation ratio was 1:10 (PA04:PA37), AFB_1_ inhibition was 39.10% (Figure 5), and its AFB_1_ content was 1024.1 ng/mL (Figure 4). Inoculum containing PA10 had similar results on peanuts. When the inoculation ratio was 10:1 (PA10:NRRL3357), the lowest detected AFB_1_ content occurred 1067.1 ng/mL (Figure 4) and the AFB_1_ inhibition rate was 95.05% (Figure 5). When the inoculation ratio was 1:10 (PA10:NRRL3357), AFB_1_ inhibition was 16.47% (Figure 5) and the AFB_1_ concentrations averaged 18,561.1 ng/mL (Figure 4). When the inoculation ratio was 10:1 (PA10:PA37), the lowest detected AFB_1_ content occurred 148.0 ng/mL (Figure 4) and the AFB_1_ inhibition rate was 92.51% (Figure 5). When the inoculation ratio was 1:10 (PA10:PA37), AFB_1_ inhibition was 28.16% (Figure 5) and the AFB_1_ concentrations averaged 1204.1 ng/mL (Figure 4).

After 14 days incubation, inoculations with genotype mixtures exhibited similar inhibition as that found 7 days after inoculation, with the aflatoxin content decreasing as the proportion of atoxigenic *A. flavus* increased. Using ELISA, treatments with an inoculation ratio was 10:1 (PA04:NRRL3357) had the lowest AFB_1_ content (1296.5 ng/mL; Figure S3), and the AFB_1_ inhibition rate was as high as 83.39% (Figure S4). When the inoculation ratio was 1:10 (PA04:NRRL3357), the lowest AFB_1_ inhibition rate occurred 28.46% (Figure S4), with AFB_1_ concentrations averaging 6649.8 ng/mL (Figure S3). Similarly, with PA10, a ratio of 10:1 (PA10:NRRL3357) resulted in the lowest AFB_1_ concentrations detected (1142.1 ng/mL) and AFB_1_ inhibition averaged 87.72%. However, when the inoculation ratio was 1:10 (PA04:NRRL3357), aflatoxin biosynthesis was only slightly inhibited, and the AFB_1_ inhibition rate was only 8.76% with an AFB_1_ content of 8473.6 ng/mL. Aflatoxin concentrations measured with TLC provided similar results (Figure S5).

Competition on PDA yielded similar results to competition on peanuts, only with less variability. As the proportion of the spore mixture composed of either PA04 or PA10 increased, inhibition of aflatoxin biosynthesis increased (Figure 3). However, percent inhibition on agar was less than that observed on peanuts (Figure 5). The lowest mean AFB_1_ concentration was 4947.0 ng/mL and the highest was 18,187.4 ng/mL when NRRL3357 was mixed with PA04. The lowest AFB_1_ inhibition rate was 2.96% and the highest AFB_1_ inhibition rate was 73.60%. When PA10 was mixed with NRRL3357, the lowest level of AFB_1_ was 7922.3 ng/mL and the highest level was 18,212.3 ng/mL, with the lowest rate of AFB_1_ inhibition being 2.82% and the highest inhibition 57.73%. Overall, inhibition of aflatoxin biosynthesis by the two atoxigenic strains PA04 and PA10 on PDA was less than on peanuts.

## 4. Discussion

Shandong Province, one of the largest peanut-producing areas in China, has a long history of peanut aflatoxin contamination. This contamination severely limits economic development. Dorner et al. [32] found that spraying field plots with a mixture of atoxigenic *A. flavus* and atoxigenic *A. parasiticus* reduced pre-harvest aflatoxin contamination of peanuts, and also had a beneficial legacy effect of reducing aflatoxin contamination during storage. Since that time several atoxigenic strain-based granular products have been developed for use on peanuts and are in use on commercial peanuts in the US, Senegal, Nigeria, and the Gambia [33,34,35]. The use of native atoxigenic *A. flavus* as a biocontrol agent to prevent aflatoxin contamination has several advantages including low environmental impact. Atoxigenic strains theoretically have similar adaptations and environmental competence as aflatoxin producers and atoxigenic strains are equally effective at colonizing and utilizing crop tissues [10]. It has repeatedly been suggested that native endemic atoxigenic genotypes are superior to introduced genotypes [36] due to better adaptation to the target agroecosystem and associated niches. In addition, the use of endemic fungi reduces the risk of any potential detrimental influences on the environment and non-target hosts and avoids potential unforeseen and undesirable outcomes from biological invasions [37,38].

In this study, two atoxigenic L strain morphotype *A. flavus* genotypes PA04 and PA10 were obtained from peanut kernels collected in Shandong Province. When co-inoculated with *A. flavus* NRRL3357 and local aflatoxin-producer PA37, these atoxigenic genotypes displayed superior competitiveness. Generally, lower concentrations of aflatoxins are produced by L strain *A. flavus* compared to S strain *A. flavus* [12,39]. Some L morphotype *A. flavus* lack ability to produce aflatoxin [11] and some of the molecular events that led to atoxigenicity occurred many thousands of years BPE [40]. In addition, L strain *A. flavus* produce large numbers of conidia which are advantageous in airborne transmission and crop establishment [41]. The frequency of *A. flavus* genotypes in communities of aflatoxin-producing fungi is determined in part by the number of conidia and this frequency has an impact on the level of crop aflatoxin contamination [42].

The two atoxigenic *A. flavus* genotypes, PA04 and PA10, identified in the current study, are native to Shandong Province, and both reduce aflatoxin levels in peanuts and on PDA media (Figure 4). The extent to which aflatoxin biosynthesis is inhibited is correlated with the proportion of atoxigenic strain conidia present. These results suggest that both of these atoxigenic *A. flavus* have the potential for biological control of aflatoxin contamination of peanuts in Shandong Province. The sclerotia of *A. flavus* is an important survival structure in soil and plant residues. Sclerotia generate conidia when germinating under favorable conditions. Thus, the production of sclerotia provides *A. flavus* increased potential to overwinter [43].

However, PA04 and PA10 produce both abundant sclerotia and spores. Both PA04 and PA10 are good candidates either individually or in combination for active ingredients of biocontrol products directed at managing aflatoxins in China. The two atoxigenic *A. flavus* were more effective at inhibiting aflatoxin biosynthesis on peanuts than on PDA. Superior competitiveness on peanuts may have increased the likelihood that these genotypes would be isolated from Shandong peanuts in the current study. In the field, applied biocontrol strains must be competitive against aflatoxin-producers during dispersal and crop colonization. Meanwhile, the formulation of biocontrol fungi must be able to effectively deliver the necessary number of conidia to gain a competitive advantage at an acceptable cost [27]. Therefore, field studies on the efficacy and persistence of these two atoxigenic strains of *A. flavus* under standard agronomic practices in the target areas are necessary [44]. Currently, the most practical method for delivering atoxigenic stains for aflatoxin management is to combine the atoxigenic fungi with a food source, usually a grain or a grain byproduct. After application, the fungus utilizes the nutrients in the formulation to support mycelial growth and spore formation [27]. To protect crops from aflatoxin contamination in the long term, the natural diversity of aflatoxin populations in agricultural soils needs to be further understood and optimal practices to utilize atoxigenic *A. flavus* as biocontrol agents should be explored [45].

It is important to limit potential risks associated with biocontrol including prevention of crop damage and damage to the soil microbiota. Atoxigenic strains have been used to greatly reduce aflatoxin-contamination in commercial agriculture since 1996 without observed detrimental effects [23,27]. Atoxigenic *A. flavus* strains PA04 and PA10, identified in the current study, are highly promising candidate fungi for active ingredients for biocontrol products directed at preventing aflatoxin contamination in Shandong Province, China. The potential of these fungi and the track record of atoxigenic strain-based biocontrol provide an opportunity to improve both the prosperity of Shandong agriculture and the health of the population that consumes Shandong-produced peanuts. Field trials directed at evaluating and optimizing the use of PA04 and PA10 in Shandong are needed.

## Supplementary Materials

The following are available online at https://www.mdpi.com/article/10.3390/jof7090773/s1. Figure S1: The electrophoretogram of 14 strains of *Aspergillus flavus* were selected from different samples and different batches; Figure S2: The CPA content of *A. flavus* PA04 and PA10 was determined by thin layer chromatography (TLC); Figure S3: Determination of AFB_1_ content in peanut cultured for 14 d by ELISA; Figure S4: Inhibition of AFB_1_ biosynthesis by NRRL 3357 with co-inoculation of different proportions of atoxigenic strains on peanuts at 30 °C for 14 d; Figure S5: Determination of aflatoxin content in peanut cultured for 14 d by TLC; Table S1: Reduction of AFB_1_ content by co-inoculation with atoxigenic *A. flavus*.
