# Supplementary material for: Selection of Atoxigenic Aspergillus flavus for Potential Use in Aflatoxin Prevention in Shandong Province, China
Source: J Fungi (Basel). 2021 Sep 18;7(9):773. doi: 10.3390/jof7090773 (PMC8472152; doi:10.3390/jof7090773)
Supplement: Supplementary file 1 [file jof-07-00773-s001.zip › jof-1268006-supplementary/Table S1.pdf]

Table S1. Reduction of AFB_1 content by co-inoculation with atoxigenic *A.flavus*

Strains	Peanut (7 d)	Peanut (14 d)	PDA (10 d)
	AFB_1 (ng/mL)	AFB_1 (ng/mL)	AFB_1 (ng/mL)
NRRL3357[a]	21560.7 ± 2971.5	9295.5 ± 683.1	18741.4 ± 500.7
PA04: NRRL3357[b]	17209.6 ± 3276.0	6649.8 ± 910.3	18187.4 ± 492.7
PA04: NRRL3357[c]	8517.8 ± 1467.6	2834.0 ± 104.5	16450.0 ± 968.7
PA04: NRRL3357[d]	923.1 ± 889.4	1296.5 ± 53.2	4947.0 ± 364.6
PA10: NRRL3357[b]	18561.1 ± 2039.8	8473.6 ± 432.5	18212.3 ± 294.3
PA10: NRRL3357[c]	17322.4 ± 1334.0	3317.7 ± 105.2	17252.7 ± 629.5
PA10: NRRL3357[d]	1067.1 ± 365.1	1142.1 ± 246.1	7922.3 ± 2058.0
PA37[a]	1976.7 ± 540.2		
PA04: PA37[b]	1204.1 ± 398.1		
PA04: PA37[c]	825.3 ± 288.1		
PA04: PA37[d]	171.7 ± 29.9		
PA10: PA37[b]	1420.3 ± 203.1		
PA10: PA37[c]	1082.5 ± 136.9		
PA10: PA37[d]	148.0 ± 44.6		

[a] Positive control whereby values are based solely on AFB_1 concentration of NRRL3357/PA37

[b] The spore concentration ratio is 1: 10

[c] The spore concentration ratio is 1: 1

[d] The spore concentration ratio is 10: 1
