# Supplementary figures and images for: Selection of Atoxigenic Aspergillus flavus for Potential Use in Aflatoxin Prevention in Shandong Province, China
Source: J Fungi (Basel). 2021 Sep 18;7(9):773. doi: 10.3390/jof7090773 (PMC8472152; doi:10.3390/jof7090773)

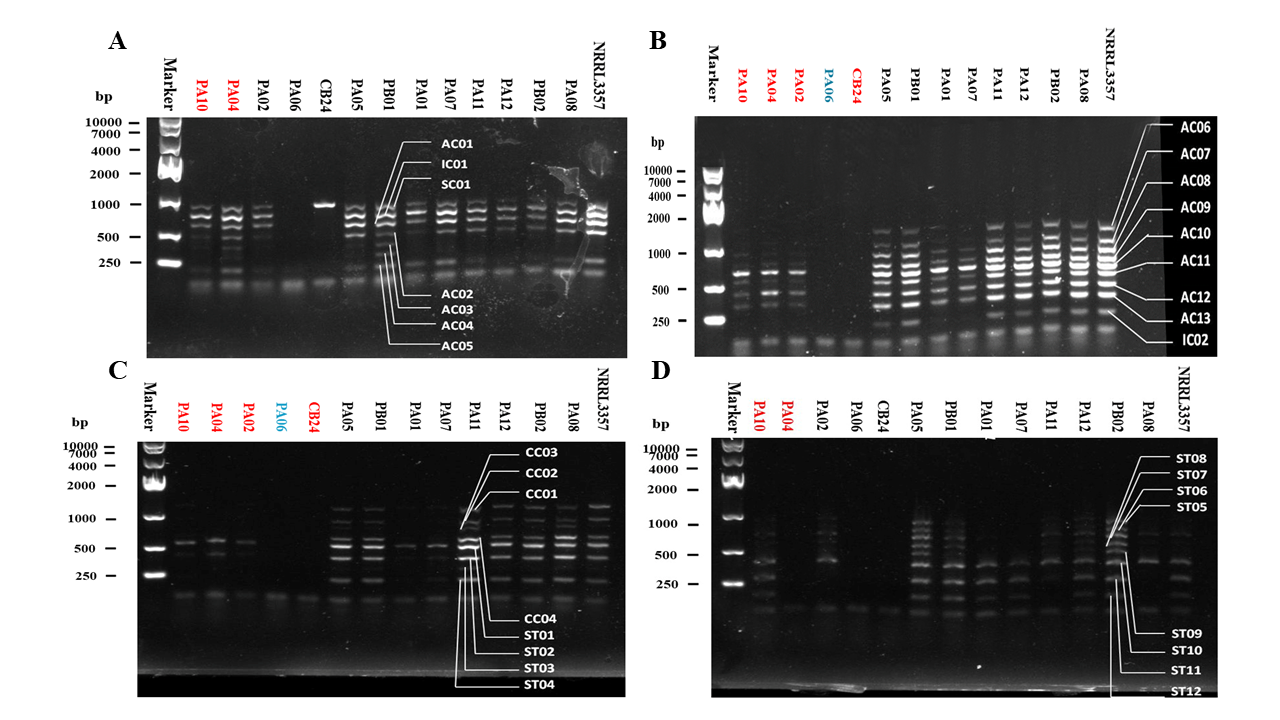

Supplement: Supplementary file 1 [file jof-07-00773-s001.zip › jof-1268006-supplementary/Fig.S1.tif]

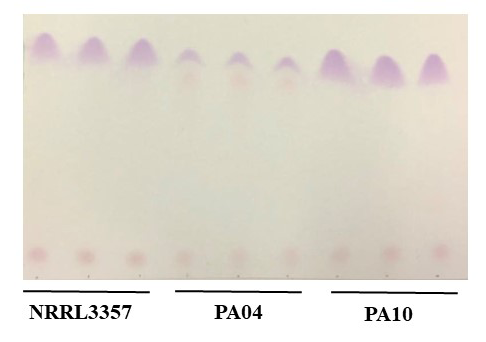

Supplement: Supplementary file 1 [file jof-07-00773-s001.zip › jof-1268006-supplementary/Fig.S2.tif]

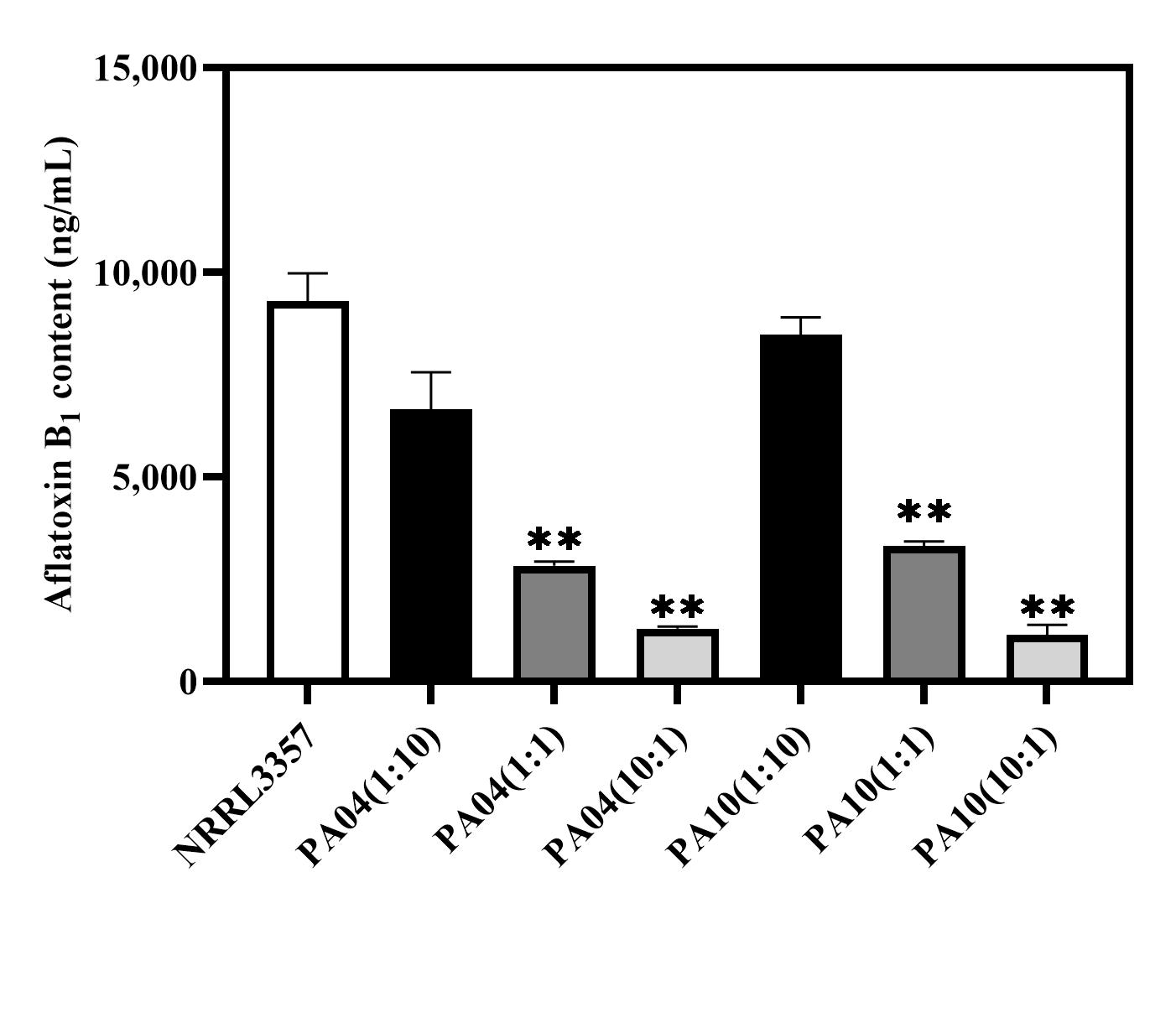

Supplement: Supplementary file 1 [file jof-07-00773-s001.zip › jof-1268006-supplementary/Fig.S3.jpg]

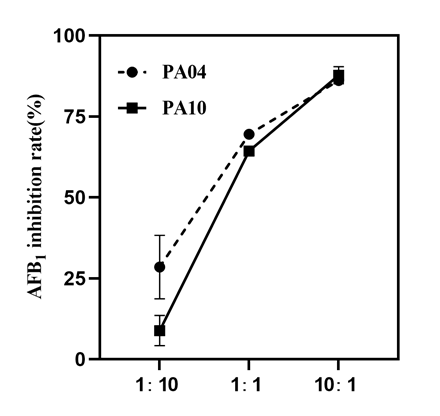

Supplement: Supplementary file 1 [file jof-07-00773-s001.zip › jof-1268006-supplementary/Fig.S4.tif]

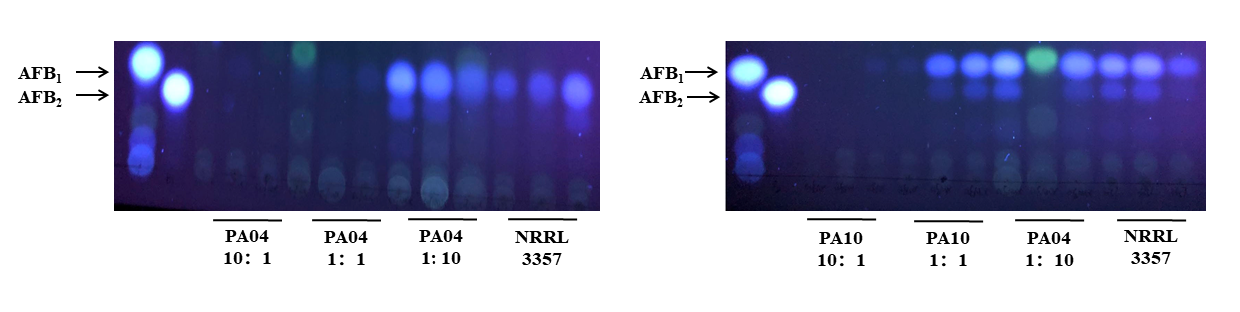

Supplement: Supplementary file 1 [file jof-07-00773-s001.zip › jof-1268006-supplementary/Fig.S5.tif]
